# Identification and binding mode of a novel *Leishmania* Trypanothione reductase inhibitor from high throughput screening

**DOI:** 10.1371/journal.pntd.0006969

**Published:** 2018-11-26

**Authors:** Lorenzo Turcano, Esther Torrente, Antonino Missineo, Matteo Andreini, Marina Gramiccia, Trentina Di Muccio, Ilaria Genovese, Annarita Fiorillo, Steven Harper, Alberto Bresciani, Gianni Colotti, Andrea Ilari

**Affiliations:** 1 IRBM Science Park S.p.A. Via Pontina Km 30,600–00071 Pomezia (RM), Italy; 2 Dipartimento di Malattie Infettive,—Istituto Superiore di Sanità Viale Regina Elena, Roma, Italy; 3 Dipartimento di Scienze Biochimiche, “Sapienza” Università di Roma P.le A. Moro, Roma, Italy; 4 Istituto di Biologia e Patologia Molecolari–CNR, and Dipartimento di Scienze Biochimiche, “Sapienza” Università di Roma P.le A. Moro, Roma, Italy; Drexel University, UNITED STATES

## Abstract

Trypanothione reductase (TR) is considered to be one of the best targets to find new drugs against Leishmaniasis. This enzyme is fundamental for parasite survival in the host since it reduces trypanothione, a molecule used by the tryparedoxin/tryparedoxin peroxidase system of *Leishmania* to neutralize hydrogen peroxide produced by host macrophages during infection. In order to identify new lead compounds against *Leishmania* we developed and validated a new luminescence-based high-throughput screening (HTS) assay that allowed us to screen a library of 120,000 compounds. We identified a novel chemical class of TR inhibitors, able to kill parasites with an IC_50_ in the low micromolar range. The X-ray crystal structure of TR in complex with a compound from this class (compound **3**) allowed the identification of its binding site in a pocket at the entrance of the NADPH binding site. Since the binding site of compound **3** identified by the X-ray structure is unique, and is not present in human homologs such as glutathione reductase (hGR), it represents a new target for drug discovery efforts.

## Author summary

Human leishmaniasis is one of the most diffused neglected vector-borne diseases and causes 60,000 deaths annually, a rate surpassed only by malaria among parasitic diseases. Anti-*Leishmania* treatments are unsatisfactory in terms of their safety and efficacy and there is an urgent need to find treatments. Compounds targeting proteins that are essential for parasite survival but that are not present in the human host are of especial interest with a view to developing selective and non-toxic drugs. *Leishmania* uses trypanothione as its main detoxifying molecule, allowing the parasite to neutralize the reactive oxygen species produced by macrophages during the infection. Trypanothione is activated by Trypanothione reductase (TR), an enzyme that is absent in man but that is essential for parasite survival, and is therefore considered an attractive target.

The new luminescence-based high-throughput screening assay that we have developed and validated allowed us to identify new TR inhibitors by screening a collection of 120,000 compounds. Hit follow-up led to a prototype molecule, compound **3,** that we have shown is able to bind in a cavity at the entrance of the NADPH binding site, thereby inhibiting TR. Compound **3** is not able to inhibit the human homolog glutathione reductase (hGR) since the residues lining its NADPH binding cavity are not conserved with respect to TR. Based on their mechanism of action, compounds from the class represented by compound 3 are useful leads for the design new drugs against leishmaniasis.

## Introduction

Protozoan parasites from the *Leishmania* genus are the causative agent of leishmaniasis, a neglected tropical disease that infects numerous mammals (including humans) throughout the world. Human leishmaniasis consists of three major clinical forms: visceral leishmaniasis (VL), which is fatal if left untreated; cutaneous leishmaniasis, which can heal spontaneously but leaves disfiguring scars; and mucocutaneous leishmaniasis, which is not self-healing and can potentially be fatal. VL is caused by *Leishmania donovani* in East Africa and the Indian subcontinent and by *Leishmania infantum* in Europe, North Africa, and Latin America. VL causes an estimated 60,000 deaths annually, a rate surpassed among parasitic diseases only by malaria.[1, 2] *Leishmania* spp., together with other protozoan pathogens responsible for diseases such as sleeping sickness (*Trypanosoma brucei*) and Chagas disease (*Trypanosoma cruzi*), belong to the Trypanosomatidae (trypanosomatids) family. The therapy of Trypanosomatidae, and in particular of VL, represents a neglected area of research and drug discovery/development. Currently, treatments for these diseases (e.g. antimony, amphotericin, paramomycin, miltefosine, aminoquinoline, sitamaquine) are unsatisfactory in terms of their safety and efficacy.[3] This sharply contrasts with the therapeutic need for VL treatments, number of affected patients, and associated fatalities. This discrepancy is primarily due to the distribution of these infections in neglected tropical and subtropical geographies that have inadequate research capacities and public-health infrastructures. Advances in the field of leishmaniasis drug discovery, recently reviewed by Zulfiqar and colleagues[4] suggest that new compounds targeting proteins essential for parasite survival but that are absent in the human host are attractive targets when demonstrated to be translatable *in vivo*.

**Fig 1:**
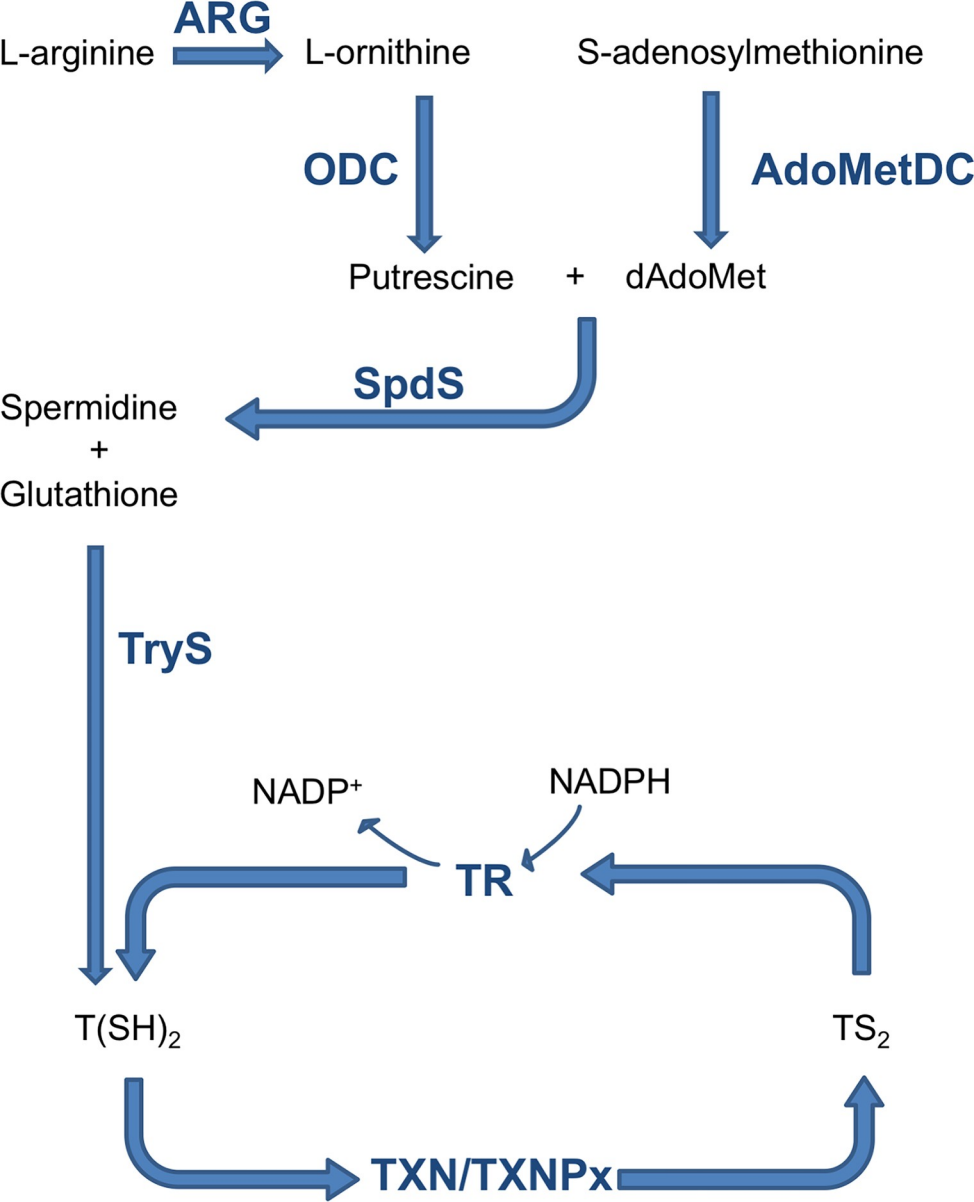
Schematic representation of the trypanothione pathway.

In contrast to the mammalian redox defense machinery that is based on glutathione, trypanosomatid parasites possess trypanothione as the main detoxifying system against oxidative damage. Trypanothione [N1,N8-bis(glutathionyl)spermidine] (TS_2_), which is synthesized by trypanothione synthetase (TryS) and reduced to T(SH)_2_ by the trypanothione reductase (TR), is used by the couple tryparedoxin/tryparedoxin peroxidase I (TXN/TXNPx) to neutralize hydrogen peroxide produced by macrophages during infection (**Fig 1**).[5–7] All attempts to date to obtain a TR-null mutant in *L*. *donovani* have failed and mutants displaying a partial trisomy of TR locus, where two TR alleles are disrupted by gene targeting, show attenuated infectivity and decreased ability to survive within the macrophages.[8] TR is thus an attractive target for the development of potential new drugs against trypanosomatids since it is essential for parasite survival but is absent in the human host. The human homolog, glutathione reductase (hGR), that maintains glutathione in a reduced state, has a significantly different substrate binding site in terms of both volume and charge distribution, suggesting the possibility of identifying molecules able to selectively target TR.[5]

The crystal structure of *L*. *infantum* TR[9] has been shown to be very similar to the structure of other trypanosomatid TRs solved to date such as those from *Crithidia fasciculata*, *T*. *cruzi* and *T*. *brucei*.[10–12] TR is a two-fold symmetrical homodimer in which each subunit is formed by three domains, the interface domain (residues 361–488), the NADPH binding domain (residues 161–288) and the FAD binding domain (residues 1–160 and 289–360). The trypanothione binding site resides in a large cavity at the interface between the two monomers, formed by the residues of the FAD binding domain of one sub-unit and those of the interface domain of the other. Reduction of trypanothione catalyzed by TR takes place by a mechanism in which two electrons are transferred from NADPH via FAD to the Cys52-Cys57 disulfide bridge. TS_2_ then binds to the protein and Cys52, deprotonated by the couple His461'-Glu466', nucleophilically attacks the trypanothione disulfide bridge resulting in formation of a mixed disulfide. Finally, the attack of Cys57 on Cys52 promotes the release of the reduced substrate.

Metals and metal-containing compounds can inhibit TR by binding to the active site;[13–16] and antimony-containing drugs that block TR activity are known.[9] In addition to such metal compounds that bind to the two active catalytic cysteines,[17] several organic compounds have also been shown to inhibit TR activity. Diaryl sulfide,[18, 19] 2-iminobenzimidazoles, [20] polyamine analogs, [21] quinoline-based compounds,[22] pyrimidopyridazine-based scaffolds,[22] azole-based compounds[23] and lunarine analogs[24] have all been reported. Some of these compounds were identified by high throughput screening (HTS) employing the colorimetric method for detection of TR activity [22, 25] developed by Hamilton *et al*.[26]

In the current work our studies aimed first at setting up a new assay in which NADPH oxidation was coupled to a luminescence assay. After optimization for screening, the assay was used in an HTS effort to identify new classes of compounds able to inhibit TR and to act against *Leishmania* parasites. For this purpose, we screened our in-house library containing 120,000 compounds and succeeded in identifying a novel hit series of *L*. *infantum* TR inhibitors. Compound **3**, a representative compound from this new series of TR inhibitors, was shown by X-ray crystallography to bind at previously unknown but druggable site at the entrance of the NADPH binding cavity. Compounds from this class of TR inhibitors were shown to have activity against *Leishmania* parasites in a growth assay.

## Methods

### Compound collection

At the time the HTS was performed, a collection of approximately 120,000 small molecules from both commercial and non-commercial suppliers was available through the CNCCS public-private consortium (www.cnccs.it). In addition to FDA and/or EMA approved drugs the collection contained a structurally diverse range of chemotypes with average molecular weight 370 Daltons. The collection was constructed aiming for high internal structural diversity (average Tanimoto distance from the nearest neighbour of 0.38) [27] while maintaining an attractive distribution of physicochemical properties (e.g. calculated logD, sp3 character, hydrogen bond donor/acceptors and total polar surface area).

### Enzymatic assay

*L*. *infantum* TR was cloned and purified as previously described.[9, 28] Bovine serum albumin (BSA), NADPH, auranofin, hGR, oxidized glutathione (GSSG) and DTNB were purchased from Sigma-Aldrich (St. Louis, MO); oxidized trypanothione (TS_2_) was purchased from Bachem (Bubendorf, Switzerland); the NADPH-Glo kit was purchased from Promega (Madison, WI, U.S.A.). Compounds, dissolved in DMSO, were transferred to 384-well white plates (Greiner Bio One, Frickenhausen, Germany) using the acoustic droplet ejection technology (ATS-100, EDC Biosystems, U.S.A.) to reach the desired final concentration. The TR enzymatic reaction was performed by addition of 0.1 nM TR, 12.5 μM NAPDH, 15 μM TS_2_ in 50 mM HEPES (pH 7.4), 40 mM NaCl, 0.01% BSA in a final volume of 15 μL. After 60 min of incubation at room temperature, an equal volume of NADPH-Glo reagent was added and the luminescent signal was acquired by an EnVision plate reader (PerkinElmer, Waltham, MA, U.S.A.). In fact the NADPH-Glo signal was demonstrated to stabilize after 30 minute (**S1 Fig**) according to the reagent data sheet. In addition, in order to make sure that the production of a reducing agent (i.e. T(SH)_2_) by the TR activity was not going to interfere with the assay, and in absence of T(SH)_2_ as purified reagent, the effect of DTT and GSH was tested on the NADPH-Glo reaction resulting in no interference at concentrations relevant for the current setup (**S2 Fig**). The hGR assay was performed by addition of 0.5 nM hGR, 10 μM GSSG, 20 μM NADPH in 50 mM HEPES (pH 7.4), 40 mM NaCl, 0.01% BSA in a final volume of 15 μL. After 30 min incubation at room temperature 15 μl of NADPH-Glo kit was added to reveal the signal. The 5,5′-dithiobis(2-nitrobenzoic acid) (DTNB) assay was performed using 2 nM TR, 100 μM NADPH, 4 μM TS_2_ and 200 μM DTNB in 40 mM HEPES (pH 7.4), 1 mM EDTA, 0.01% BSA and 0.05% tween-20 in a final volume of 50 μl. The absorbance signal (412 nm) was acquired 15 minutes post incubation at room temperature using the plate reader (Safire2, Tecan, Switzerland). Results were analyzed using Prism software (GraphPad, San Diego, CA, U.S.A.) and Vortex (Dotmatics, Bioshops Stortford, UK). Dose-response curves were fitted by four-parameter logistic regression.

### Compound similarity search

After hit confirmation compound similarity searches were performed by generation of circular Morgan fingerprints (radius 2, 2048 bits) for the test compounds using open source RDKit software (http://www.rdkit.org/, release 2014_09_2). The molecular representations generated were used to perform ligand based virtual screening against the target databases (i.e. our own screening collection) that is described above or a subset of the public ZINC database (http://zinc.docking.org) from which PAIN’s and undesirable structures were removed). Similarity was assessed by the Tanimoto index between the reference and target structures using a cut-off of 0.6. Similar compounds were clustered using Taylor-Butina clustering, a non-hierarchical clustering method that ensures that each cluster contains molecules with a set cut-off (or threshold) distance from a central compound.[29] Compounds selected for purchase or screening follow up were chosen from the most populated clusters, with either the central compound or a close analog (based on visual inspection) being used to represent the compound cluster. All selected compounds were quality controlled by UPLC-MS prior to testing.

### Competition assay

Competition assays were performed using three test concentrations (4, 16 and 64 μM) for the competing compounds. The reaction buffer and components were mostly identical to those used in the TR assay described above, but 1 nM TR was employed for 5 minutes against a serial dilution of either TS_2_ in presence of 40 μM NADPH or NADPH in presence of 30 μM TS_2_. The residual amount of NADPH was measured using the NADPH-Glo kit (Promega, U.S.A.) after 30 min incubation at room temperature as per the manufacturer’s protocol. The luminescent signal was measured using the EnVision plate reader (PerkinElmer, USA). IC_50_, V_max_, and *K*_m_ values were calculated using Prism software (GraphPad, San Diego, CA, U.S.A.).

### Binding assay by SPR

Surface plasmon resonance (SPR) interaction analysis was performed using a Biacore T200 (GE Healthcare, Uppsala, Sweden). TR was immobilized on a CM4 chip by amine coupling according to manufacturer’s instructions (Amine Coupling Kit, GE Healthcare, Uppsala, Sweden). Briefly, the surface of the sensor chip was activated for 7 min using a mixture of 0.1 M *N*-hydroxy succinimide (NHS) and 0.4 M *N*-ethyl-*N*’-[3-dimethyl-aminopropyl] carbodiimide (EDC) then 25 μg/mL of TR in 10 mM sodium acetate (pH 5.0) was injected for 360 s at 10 μl/min, finally residual activated groups on the surface were blocked by a 7-min injection of 1 M ethanolamine (pH 8.5). A reference channel for background subtraction was prepared by activation with EDC/NHS mixture (0.1 M/0.4 M as per ligand immobilization), followed by blocking with 1 M ethanolamine. The binding of the selected hit to the immobilized ligand was evaluated by a multi-cycle kinetic procedure in 50 mM HEPES pH 7.4, 40 mM NaCl, 0.05% Tween-20 supplemented with 2% DMSO (Sigma Aldrich). The analyte was injected for 20 s at 30 μl/min until equilibrium and dissociation monitored for 120 s. A standard curve of DMSO was included for solvent correction. Biomolecular binding events were reported as changes of resonance units (RUs) over time. The data were analysed by the Biacore T200 evaluation software: specific sensorgrams were obtained for the analyte, by subtracting the signals of the reference channel to those of the TR-immobilized one, and corrected for DMSO interference from DMSO standard curve. The binding affinity was evaluated at steady state whereas the kinetic parameters were calculated by a one-phase association and two-phase dissociation model using the Prism software (GraphPad, San Diego, CA, U.S.A.)

### X-ray structure determination

*L*. *infantum* TR was cloned, purified and crystallized as previously reported.[9, 28] The crystals were obtained by the hanging drop vapor diffusion method at a temperature of 294 K, using as precipitating agent ammonium sulfate in a range between 1.4 and 1.7 M, in a Tris-HCl buffer at pH 8.0–8.7. The crystals reached a dimension of 0.3 mm x 0.3 mm x 0.2 mm in about 7 days. Crystals of the native oxidized TR were soaked for 2 h using a stabilizing solution containing 1.5 M ammonium sulfate, 0.1 M Tris pH 8.5 and 1 mM compound **3** (2-(diethylamino)ethyl-4-((3-(4-nitrophenyl)-3-oxopropyl)amino)benzoate). Crystals were cryo-protected in a solution of mother liquor 75% and glycerol (25%), then flash-frozen by quick submersion into liquid nitrogen for transport to a synchrotron radiation source.

Single wavelength data sets (λ = 1.073 Å) were collected at the beamline ID29 at the Synchrotron Radiation Source ESRF, Grenoble (France) using a Dectris Pilatus 6M-F detector at a temperature of 100 K. The data sets were processed and scaled with XDS.[30] The crystals belong to the P4_1_2_1_2 space group with the following cell dimensions: a = 103.33 Å, b = 103.33 Å, c = 191.55 Å. Crystal parameters and complete data collection statistics are reported in **Table 1**. The structure of *L*. *infantum* TR in complex with compound **3** was solved with the program Molrep [31] by molecular replacement using the native TR (PDB code: 2JK6) [9] as search model. Refinement was performed using the program REFMAC5[32] and model building was carried out using the program COOT.[33] The structure of TR in complex with compound **3** was refined to a final free R value of 20.6% for all resolution shells (73–3.37 Å), calculated using the working set reflections (14626), and Free R value, calculated using the test set reflections (737), of 26.5%. The final model is a monomer containing 488 residues, a FAD molecule, two sulfate molecules, and one compound **3** molecule. The most favored regions of the Ramachandran plot contain 90.0% of residues, the allowed regions contain 9.0% of residues, with 1% in disallowed region. The X-ray structure has been deposited in the protein data bank with the PDB (Protein Data Bank) code 6ER5.

**Table 1:** Crystal parameters, data collection statistics and refinement statistics of compound 3-TR complex.

Space group	P4_1_2_1_2
Unit cell parameters	
*a* (Å)	103.33
*b* (Å)	103.33
*c* (Å)	191.55
Number of molecule in the asymmetric unit	1
Wilson plot B factor (Å^2^)	118.4
**Data statistics**	
Resolution range	3.37–73.01 (3.37–3.45)
Unique reflections	15365 (1114)
Completeness	99.9 (99.6)
Redundancy	25.38 (26.29)
CC1/2	99.9 (94.2)
I/σ(I)	16.4 (2.4)
**Structure Refinement**	
Resolution range	3.37–73.01 (3.37–3.45)
Reflection in bins	14626 (1045)
R_crys_ (%)	20.6 (41.1)
R_free_(%)	26.5 (37.9)
Rms bonds (Å)	0.008
Rms angle (°)	1.424
RSCC (for the compound 3)	0.58
**Ramachandran plot**	
Residues in core region (%)	90
Residues in allowed region (%)	9
Residues in generously allowed region (%)	1

Values in parentheses are for the highest-resolution shell.

### Inhibition of promastigote growth

Promastigote (the extracellular stage of the *Leishmania* life cycle) growth inhibition was evaluated using *L*. *infantum* strain (MHOM/TN/80/IPT1). To estimate the 50% inhibitory concentration (IC_50_), the MTT (3-[4.5-dimethylthiazol-2-yl]-2.5-diphenyltetrazolium bromide) micromethod[34] was used throughout the experiments with modification.[18] Promastigotes were grown in Schneider’s *Drosophila* medium (SIGMA) containing 10% Fetal Calf Serum (FCS) (GIBCO-BRL) and 2% gentamicin (50 mg/L) (Sigma) at 22°C. Parasites were adjusted to 1 × 10^6^ parasites/mL, and 200 μL of suspension was seeded in triplicate in 96-well flat bottom microplates and incubated with varying concentrations of compound. Amphotericin B (IC_50_ 0.5 μM) (Euroclone) was used as control. Each experiment was done in triplicate for each drug concentration and two independent experiments were performed. After 72 h of incubation, 30 μL of MTT was added to each well and plates were further incubated for 2 h. The absorbance at 550 nm was measured with a 96-well scanner (DINEX Technologies, VA, USA). Anti-leishmanial activity per experimental point was expressed as mean percentage of inhibition with respect to vehicle treated parasites (0%). In addition, the promastigote vitality was followed by microscopic observation after 72 h. Data were analyzed and plotted by Prism (GraphPad, San Diego, USA).

### Chemistry

Compounds were obtained from commercial suppliers and were tested without further purification. Purity of final compounds were determined using mass spectrometry (MS) and ultra-high performance liquid chromatography (UPLC). UPLC-MS analyses were performed on a Waters Acquity UPLC, equipped with a diode array and a ZQ mass spectrometer, using a Waters BEH C18 column (1.7 mm, 2.1 x 50 mm). The mobile phase comprised a linear gradient of binary mixtures of H_2_O containing 0.1% formic acid (A), and MeCN containing 0.1% formic acid (B). The following linear gradient was used (A): 90% (0.1 min), 90%-0% (2.6 min), 0% (0.3 min), 0%-90% (0.1 min). The flow rate was 0.5 mL/min. The purity of final compounds was in all cases ≥95%. ^1^H NMR spectra were recorded on Bruker AV400 spectrometer operating at 400 MHz. Chemical shifts (δ) are reported in parts per million downfield from TMS and are determined using the residual (undeuterated) NMR solvent peak as an internal standard.

*2-(Diethylamino)ethyl 4-((3-(4-nitrophenyl)-3-oxopropyl)amino)benzoate* (**3**). Compound **3** was purchased as a white solid from TimTec., ^1^H NMR (400 MHz, DMSO-*d*_6_, 294 K) δ 8.51 (2H, d, *J* = 8.4 Hz), 8.36 (2H, d, *J* = 8.4 Hz), 7.85 (2H, d, *J* = 8.0 Hz), 6.76 (2H, d, *J* = 8.0 Hz), 6.73 (1H, t, *J* = 6.7 Hz), 4.36 (2H, t, *J* = 6.0 Hz), 3.61–3.40 (4H, m), 2.91–2.85 (2H, m), 2.73–2.62 (4H, m), 1.12 (6H, t, *J* = 7.1 Hz); UPLC *t*_R_ 1.23 min (peak area 96%); MS (ES^+^) 414; (M+H)^+^; m.p. 128°C.

## Results

### TR assay development and optimization

**Fig 2:**
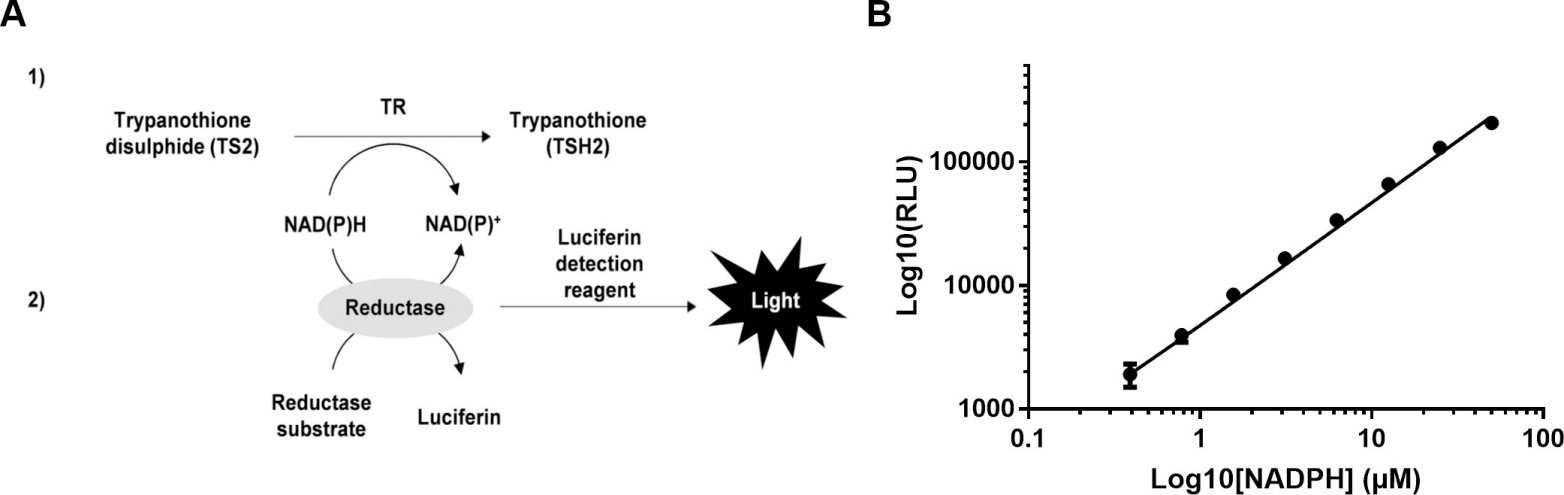
(**A**) Schematic representation of the TR assay reaction. The luciferase activity is dependent on the residual NADPH concentration after TR reaction (1). The luminescent signal, which is produced by the NADPH-Glo assay, is inversely proportional to the TR activity (2). (**B**) Sensitivity and linearity of the NADPH detection. The plot x- and y-axis are presented in logarithmic scale to allow a better appreciation of the serially diluted points. The linear correlation is plotted as a sigmoid curve on the log-log graph. The plotted points are the average of three independent replicates.

**Fig 3:**
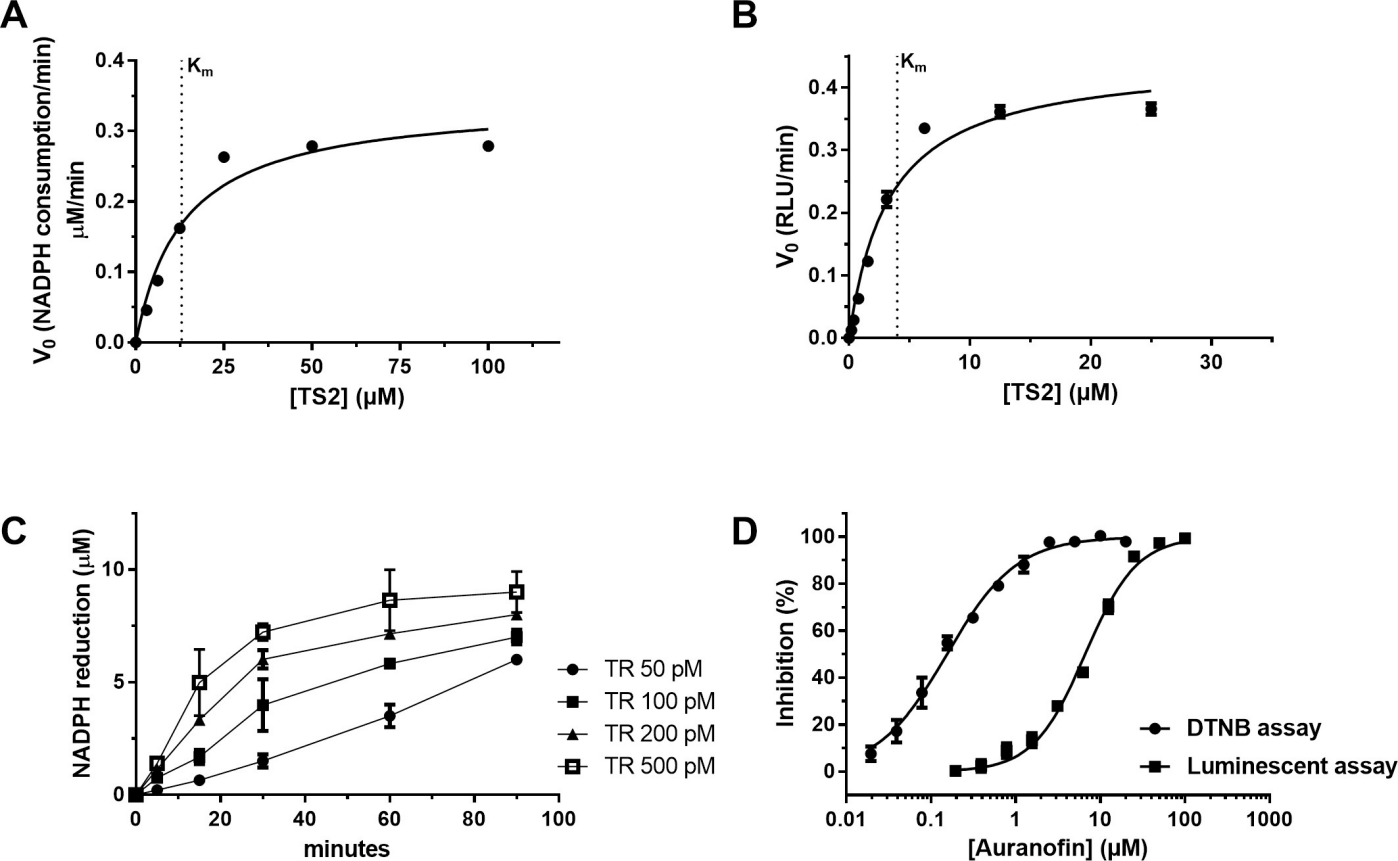
Optimization of the TR reaction. (**A**) Titration of TS_2_ in the presence of 0.5 nM TR and 12.5 μM NADPH using the luminescent assay. (**B**) Titration of TS_2_ in the presence of 20 nM TR and 100 μM of NADPH using the DTNB assay. (**C**) TR activity time course and enzyme dilution in the presence of 12.5 μM NADPH and 15 μM TS_2_. (**D**) Dose-dependent inhibition of TR activity by auranofin (AF) as determined by the DTNB and the luminescence based assay. Each experimental point is the result of three replicates (error bars bigger than the symbol size were not plotted).

Purified *Leishmania infantum* TR was used to develop a new homogeneous bioluminescent assay in which a luminescence signal is coupled to the activity of TR by reaction with the residual NADPH after reduction of TS_2_ (**Fig 2A**). This assay was miniaturized to a 384-well plate format for use in an HTS campaign. The design of the TR assay, which produces a positive signal in presence of a TR inhibitor, reduces the identification of false positives capable of interfering with luciferase activity. The linear range of the NADPH-Glo assay was established by serial dilution of NADPH, with a linear correlation between NADPH concentration and luminescent signal being appreciable up to 50 μM NADPH (**Fig 2B**). In order to optimize the substrate concentration to be used in the assay, the *K*_m_ of TS_2_ for TR was determined by titrating its saturating concentration in the presence of 0.5 nM TR and 12.5 μM NADPH. The V_0_ for each TS_2_ concentration was determined during the first 5 min of reaction and plotted against the TS_2_ concentrations. When the curve was fitted by the Michaelis-Menten equation (**Fig 3A**) the TS_2_ *K*_m_ (apparent) was found to be 13.5 ± 3.8 μM that was of the same order of magnitude of that measured by Angiulli and co-workers (K_m_ = 23 ± 1 μM).[35] To confirm this value, the *K*_m_ (apparent) of TS_2_ was determined also by the standard 5,5′-dithiobis(2-nitrobenzoic acid) (DTNB) absorbance based assay using 20 nM TR and 100 μM NADPH. The TS_2_ *K*_m_ (apparent) was found to be 3.6 ± 0.5 μM, in line with the result established by the new luminescence approach. (**Fig 3B)**. The concentration of NADPH used for further development of the luminescent assay was 12.5 μM since this value lies in the middle of the NADPH detection linearity range. Finally, an enzymatic reaction time course was carried out at four different concentrations of TR, ranging from 50 to 500 pM, keeping the TS_2_ and the NADPH concentrations constant (at 15 μM and 12.5 μM respectively). The aim of this optimization step was to identify the appropriate enzyme concentration and incubation time needed to reach a compromise with respect to (1) having the lowest possible enzyme concentration to maximize assay sensitivity, (2) identifying a time point at which a clear signal is detected over the background to minimize potential experimental variability (3) allowing a reasonable time to assemble the reaction when hundreds of microplates need to be tested and (4) ensuring that the rate of the substrate consumption is reasonably away from the reaction completion (i.e. a linear trend is appreciable between signal and time). A concentration of 100 pM TR and 60 min incubation, despite pushing the boundaries of linearity, was identified as the acceptable compromise among enzyme concentration, signal robustness and assay duration (**Fig 3C**) at room temperature. The know TR inhibitor Auranofin (AF) [15] was used to benchmark the luminescent assay’s performance with respect to the standard DTNB readout. When tested in a dose-response manner in each assay, AF’s potency was 6.6 ± 1.05 μM and 154.5 ± 10.7 nM respectively on the luminescent and DTNB assays (**Fig 3D**). This difference may be related to the known propensity of AF to form adducts with thiol bearing compounds[36] and, as a consequence, to interfere with the DTNB readout which is based on the reaction with free thiol moieties. To support to this hypothesis, a serial-dilution of AF was incubated with TR before and after the reaction took place. The signal was then developed by the addition of DTNB. The addition of AF, after the TR reaction was stopped, resulted in a dose-dependent decrease of the signal suggesting that AF does indeed at least partially interfere with the detection (**S3 Fig**).

### Hit identification

**Fig 4:**
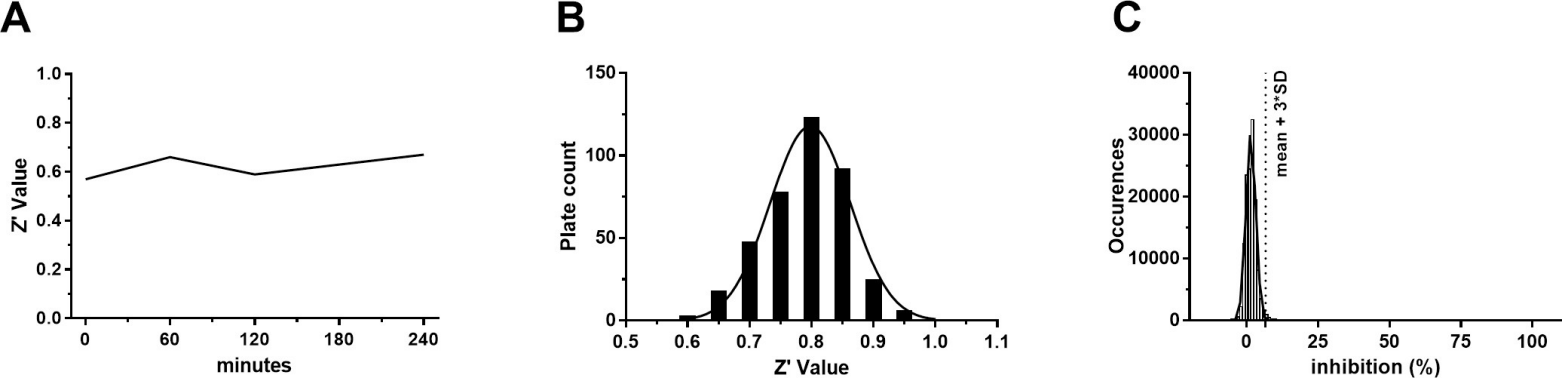
(**A**). TR activity assay Z’ time course. All components were stored at 4°C during the time course with the exception of the NADPH-Glo reagent which was kept at 22°C. (**B**) Z′ distribution of all collection plates. (**C**) Compound activity distribution reported as number of standard deviations with respect to the whole sample average and standard deviation (error bars bigger than the symbol size were not plotted).

The stability of the TR enzyme, NADPH and TS_2_ working solutions at + 4°C and the NADPH-Glo at room temperature was tested in a time course fashion. The Z’ values [37], calculated between the no-enzyme condition and the full reaction were found to be greater than 0.5 up to 4 hours (**Fig 4A**) confirming that the assay is reliable within this time frame. As a consequence, the size of a single batch of assay plates was set in order not to exceed 4 h of total operation time. A collection of around 120,000 compounds was then screened at a fixed concentration of 10 μM using the above-described TR inhibition assay. The Z′ values of all the tested plates were found to be greater than 0.5, with an average of 0.79 (**Fig 4B**). The activity of each compound was calculated as Z-score (i.e. each compound value subtracted of the whole sample average and divided by the whole sample standard deviation). The distribution of the compound activities was found to be Gaussian (**Fig 4C**); as a consequence, compounds with activity equal to or greater than three standard deviations were considered hits (i.e. compounds whose chance to be false positive is statistically less than 0.3%). 290 compounds (0.23% of the total) were identified as active in the primary screening and subjected to confirmation assays.

### Hit confirmation

**Fig 5:**
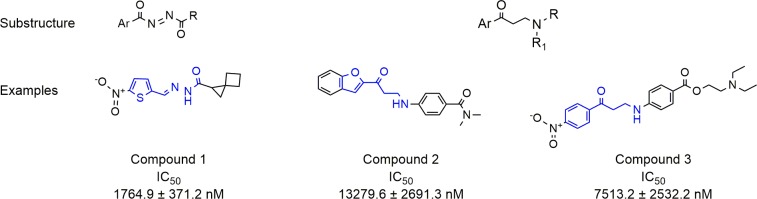
General structures of the two major hit series (substructures). Representative molecules for both series with TR inhibition potency data (examples).

In order to confirm hit compounds, dose-response curves were generated for active hits in the enzymatic assay using a starting concentration of 85 μM. TR inhibition activity was confirmed for 64 of the 290 hits identified in the primary screen with IC_50_ potencies ranging from 2 to 35 μM. As previously reported, the design of the primary assay which produces a positive luciferase signal in the presence of an inhibitor meant that no luciferase inhibition counter-screen was necessary. The 64 compounds were further prioritized into three groups: i) 22 compounds that generated curves with a slope close to 1 and that reached 100% maximum inhibition; ii) 14 compounds with slope values between 1 and 2.5 and approximately 100% maximum inhibition; iii) 28 compounds with slope close to 1 and maximum inhibition values lower than 100%. Group ii) and iii) were deprioritized due to suboptimal biochemical inhibition. From the first of these groups (group i), removal of compounds with molecular weight > 600 Daltons or by visual structural inspection, reduced the number of compounds to 15. The activity of second batches for all of these analogs was confirmed following the purchase or chemical synthesis of fresh powders. In structural terms these compounds, which were viewed as the most reliable results from the HTS effort, fell for the most part into two broad classes. Firstly, several compounds containing an aryl *N*-acylhydrazone sub-structure (such as compound **1**, **Fig 5**) were found and interestingly were conceptually similar in structure to known TR inhibitors.[23] Although compounds such as **1** showed low micromolar activity against TR they were deprioritized at this stage based on their sub-optimal structures for drug development. A small number of hits containing a 3-amino-1-aryllpropan-1-one substructure (exemplified by compounds **2** and **3** in **Fig 5**) were also found. These compounds were somewhat weaker in terms of their TR inhibition (activity in the 10–30 μM range) but were judged more suitable for follow-up. The testing of analogs around this hit series was performed with compounds being selected either from the original screening collection (potential false negatives) or through the purchase of structurally similar analogs. The selection of commercially available analolgs from the ZINC database was based initially on the presence of the required substructure and analogs were clustered based on Tanimoto similarity (threshold 0.6) using circular fingerprints. Selected analogs (about 40 compounds) were tested against TR and their specificity with respect to human glutathione reductase (hGR) inhibition was also determined. The hGR assay was performed using the same approach as the TR assay (see methods section). The results from this follow up are summarized (for active analogs only) in **S1 Table**. Interestingly, while compound **2** (one of the most active of the original hits) was not selective against hGR, follow up compounds showed a clear trend toward selective behaviour (hGR IC_50_ values were typically above 85μM). Although this follow up did not lead to strongly improved activity, the confirmation of around 25% of the selected analogs as micromolar inhibitors of TR with weak potency on hGR provided a level of comfort that these data were not false positive in nature. Based on their low molecular weight and relatively simple and tractable chemical structures compounds from the hit series were further profiled, and their interaction with TR was investigated using compound **3** (**Fig 5**) as a representative of the class. The choice of compound **3 (S4 Fig)** over other hits based on the 3-amino-1-arylpropan-1-one substructure (S1 Table) was based on it having acceptable in vitro potency (7.5 ± 2.5 μM, **S5 Fig**) that was confirmed by the DTNB assay, its high solubility (> 100 μM in assay buffer), and its relative structural complexity with respect to its analogs. The presence of a nitro group as an undesirable structural feature in **3** was mitigated by structure activity relationship data from our hit confirmation work (e.g. compounds **2**, **6**, **7** and **9**, **S1 Table**, supplementary information) that suggests that the nitro group is not a key pharmacophore element.

### Hit compound binding to TR

**Fig 6:**
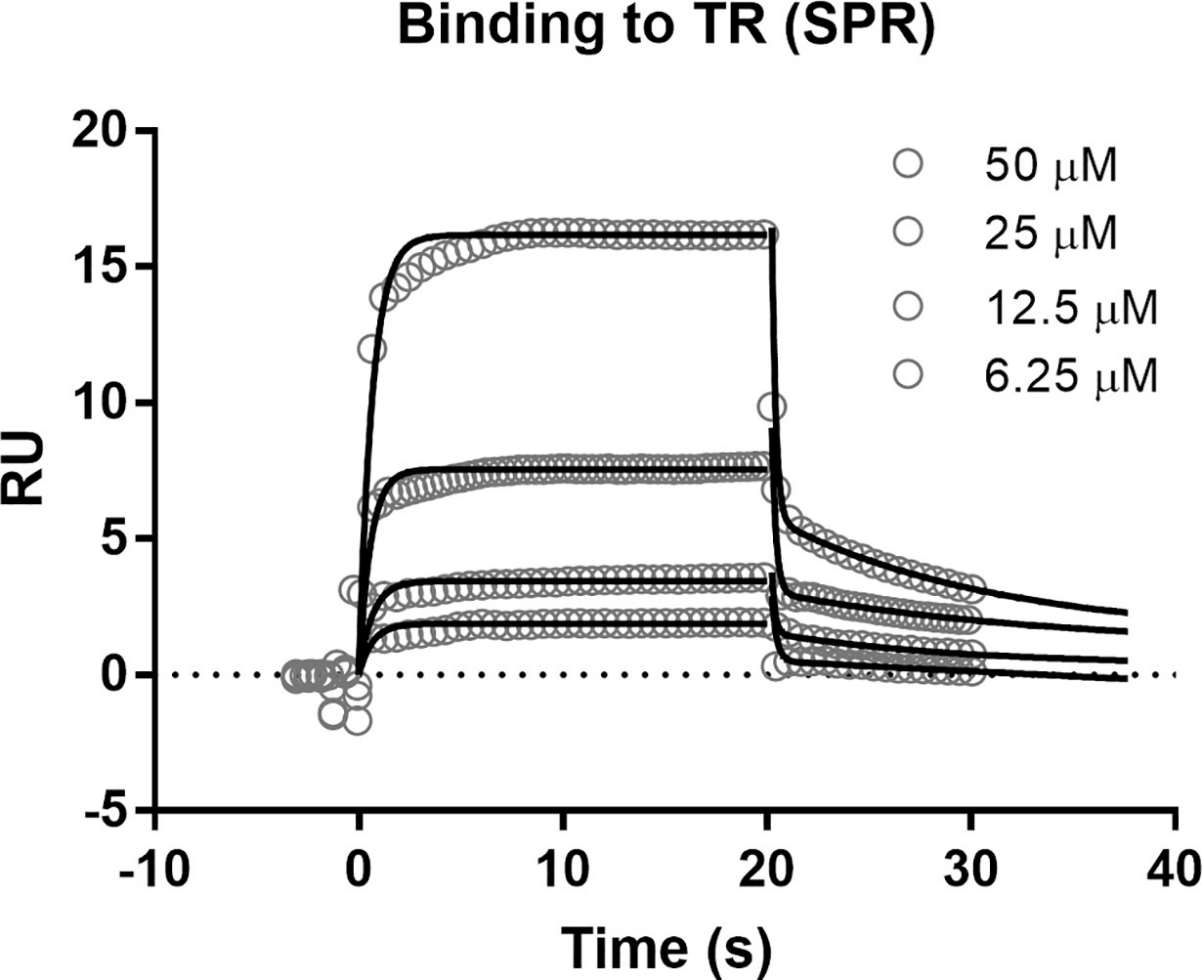
Compound 3 binding to TR as measured by SPR. Every other four experimental point is plotted to allow a cleaner representation. The plot is one representative experiment of four.

Binding of compound **3** to TR was initially evaluated by surface plasmon resonance (SPR). TR was covalently immobilized at high density (7,000 ΔRU) to a CM4 sensor chip, then the compound was injected over the surface at different concentrations, ranging from 6.25 to 50 μM. Although immobilization slightly affected the quote of active protein on chip (% TR_max_ = 18%), a reliable binding signal was achieved. The sensorgrams were best fitted with a one-phase association and two-phase dissociation model which allowed to calculate the binding kinetic parameters (*k*_on_, *k*_fast_ and *k*_slow_).Compound **3** was found to show fast on/fast off kinetics with a *k*_on_ of 3.51 ± 1.09 x 10^3^ (1/M*s), a *k*_fast_ of 5.88 ± 0.96 (1/s) and a *k*_slow_ of 0.08 ± 0.02 (1/s). The steady state calculated *K*_d_ resulted to be 28.25 ± 5.12 μM in line with the compound’s potency in the luciferase screening assay (**Fig 6**). The reported suboptimal binding kinetics are expected for a hit compound. The two phase dissociation kinetics suggest that at least part of the binding is not driven by the two molecule complementarity. Likely the *k*_slow_ is more predictive of the molecular interaction, in fact when the *k*_slow_/*k*_on_ ratio is calculated, the obtained *K*_d_ (22.7 μM) results in line with the steady state one.

Compound **3** was examined to determine whether or not it was competitive with either TS_2_ or NADPH. The activity of the TR enzyme was assayed in the presence of alternate saturating dilutions of either TS_2_ or NADPH and 4, 16 or 64 μM compound **3** (DMSO was used and negative control).

**Fig 7:**
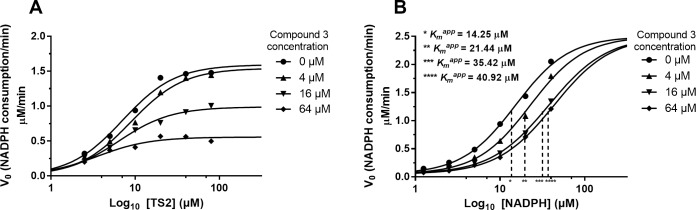
Compound 3 competition with either TS_2_ or NADPH for the TR activity. Either TS_2_ or NADPH were titrated alone or against three compound concentrations (4, 16 and 64 μM). The NADPH concentration for the TS_2_ dilutions (A) was 40 μM while the TS_2_ concentration for the NADPH dilutions (B) was 30 μM. The TR assay was performed using 1 nM TR for 5 min. Each experimental point is the average of three replicates (error bars bigger than the symbol size were not plotted).

The NADPH concentration for the TS_2_ dilutions was 40 μM while the TS_2_ concentration for the NADPH dilutions was 30 μM. Compound **3** was found be competitive for NADPH with apparent *K*_m_ shifting to the right at increasing compound concentrations. Instead, compound **3** did not alter TS_2_ *K*_m_ whereas it affected TS_2_ kinetics in terms of reaction V_max_ (**Fig 7**).

### X-ray crystal structure of TR in complex with compound 3

**Fig 8:**
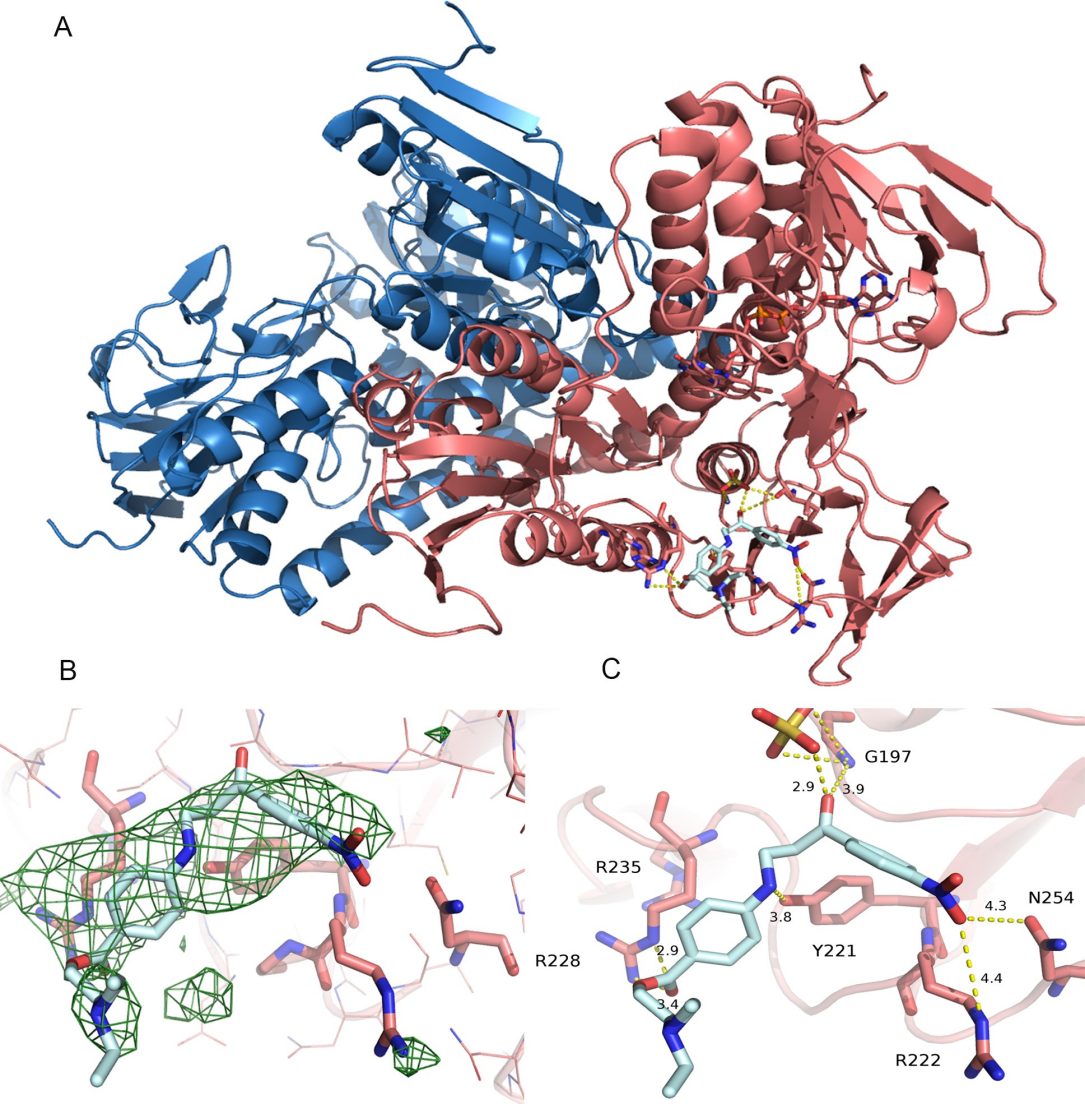
(A) Overall fold of TR in complex with compound **3**. The monomers A and B are colored in blue and magenta respectively. Compound **3** and FAD molecules are represented as sticks and colored cyan and magenta respectively. (B) Omit map (Fo-Fc) of the compound **3**-TR complex active site. The map is colored green and contoured at 3σ. (C) Blow up of the ligand binding site. The residues interacting with the ligand are indicated and represented as sticks. The picture was obtained using PyMOL (The PyMOL Molecular Graphics System, Version 2.0 Schrödinger, LLC).

In order to study the mechanism of inhibition exerted by compound **3** ((2-(diethylamino)ethyl 4-((3-(4-nitrophenyl)-3-oxopropyl)amino)benzoate)) on TR and identify the binding site of the compound X-ray crystallography studies were performed. We solved the X-ray structure of *L*. *infantum* TR in its oxidized state in complex with compound **3** (LiTR-3) at 3.37 Å resolution (**Fig 8**). The X-ray structure of the LiTR-compound **3** complex is homo-dimeric and is closely related to the TR-oxidized apo-form (**Fig 8A**).

The Fo-Fc map calculated using the dataset collected at ESRF and contoured at 3σ allowed identification of the binding site of compound **3** (**Fig 8B**). The low resolution structure and the poor quality map does not allow us to identify unequivocally the interaction of the ligand with the protein residues. However the Fo-Fc electron density map, allow the identification of the space occupied by the ligand and the residues involved in the binding even if the nitrophenyl group is not completely visible in the structure. In particular, as shown in **Fig 8C**, compound **3** is bound to the NADPH cavity entrance with an occupancy of 0.8, where it may form several mild electrostatic interactions with the protein residues lining its binding site. An electronic density peak was found close to Gly197, that has been interpreted as belonging to a sulfate ion (with 0.5 occupancy), which establishes electrostatic interactions with Gly197 and compound **3**.

As shown in Fig 8C, compound **3** is lined by Tyr221, Gly197, Asn254, Arg222 and Arg228 a residue which is electrostatically bound to Arg235 (NH1(Arg235)-NH2(Arg228) distance = 3.5 Å).

**Fig 9:**
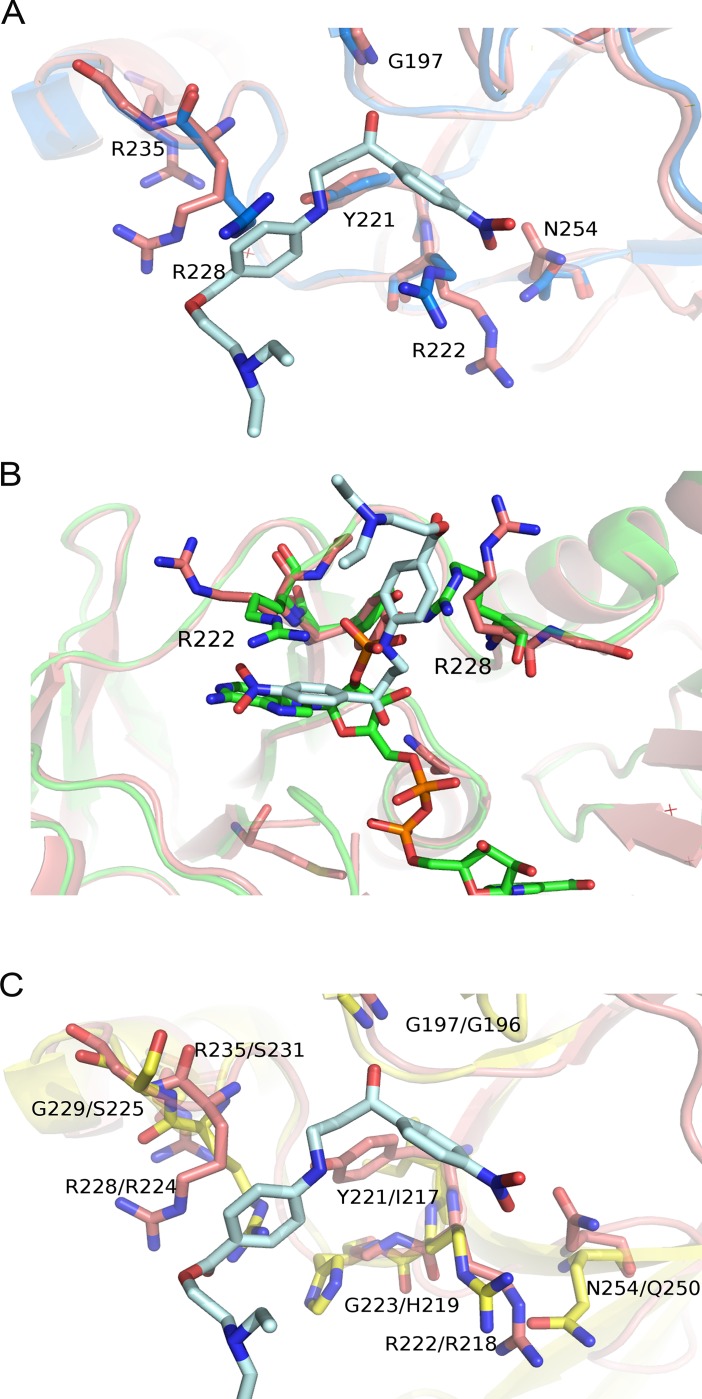
(A) Superimposition between compound **3**-TR complex and the apo TR (PDB code: 2JK6) structures. The structure of apo TR is colored blue, the compound **3**-TR complex is colored magenta. The residues involved in the ligand binding are indicated and represented as sticks. (**B**) Superimposition between compound **3**-TR complex (in magenta) and the reduced TR in complex with NADPH (in green) (PDB code: 2W0H). The two Arginine residues (Arg222 and Arg228) involved in the ligand binding are indicated and depicted as sticks. Compound **3** and NADPH are depicted as sticks and colored in light blue and green respectively (**C**) Superimposition between compound **3**-TR complex (in magenta) and Glutathione reductase (in yellow) (PDB code: 3GRS). The residues involved in the ligand binding are indicated (the residues of the compound **3**-TR complex on the left side and the residues of GR on the right side) and depicted as sticks. The picture was obtained using PyMOL (The PyMOL Molecular Graphics System, Version 2.0 Schrödinger, LLC).

The binding of compound **3** to the protein causes a local conformational change involving Arg228 and Arg222. Indeed, the superimposition between TR in oxidized state and the compound **3**-TR complex shows that Arg228 and Arg222 undergo a 30° rotation (clockwise and anticlockwise, respectively) to allow compound binding (**Fig 9A**). As shown in **Fig 9B**, the comparison between the reduced TR (PDB code 2W0H) and the compound **3-**TR complex shows that the inhibitor is placed at the entrance of the NADPH binding site. In particular, the nitro-phenyl group maps to the position of the adenine ring in the NADPH bound TR, and the two arginine residues (Arg222 and Arg228) that in the reduced enzyme interact with the adenine ribose phosphate moiety adopts a completely different conformation in the compound **3**-TR complex (**Figs 9B and S6**).

The site is unique, since it is present in TR but not in the human homologs hGR and TrxR1.

As shown by the structural analysis (**Fig 9C**), residues Tyr221 and Asn254, which are involved in ligand binding to TR, are not conserved in hGR, where they are replaced by Ile217 and Gln250, respectively. Moreover, residues Gly229 and Arg235, important for the plasticity of the site as well as the correct positioning of the substrate in the site, are substituted in hGR by residues Ser225 and Ser231. Finally, residue Gly223 is substituted in hGR by the residue His219, which changes the charge and the steric hindrance of the binding site (**Fig 9C**). The residues lining the compound-**3** binding site are not even conserved in TrxTR1; indeed the residues Arg235, Tyr221, Gly223, Gly197, Asn254 are substituted by Ala233, Val220, Ser222, Ser199, Val252 in TrxTR1 (**S7 Fig**).

The structure of the compound **3**-TR complex allows the identification of a new unique druggable site on TR which impedes the entrance of NADPH in the protein, thereby impairing trypanothione reduction. Up to now, structures of inhibitor-TR complexes have shown binding of inhibitors to the trypanothione binding site. Persch *et al*.[38] have solved the structure of *Tc*TR and *Tb*TR in complex with 5-{(5-[(1-(pyrrolidin-1-yl)cyclohexyl)]]-1,3- thiazol-2-yl]]]-1H-indole (PDB codes: 4NEW and 4NEV); Fairlamb *et al*. [12] solved the X-ray crystal structure of *Tc*TR in complex with Quinacrine mustard (*N*-*N*-{((1*S*)-4-([bis-(2-chloroethyl)amino]))-1-methylbutyl}}}*N*-(6-chloro-2-methoxy-9-acridinyl)amine} (PDB code: 1GXF); Patterson and coworkers [11] solved the X-ray structure of *Tb*TR in complex with dihydroquinazolines inhibitors (PDB codes: 2WOI, 2WOV, 2WOW, 2WP5, 2WP6, 2WPC, 2WPE, 2WPF). The two structures of TR from *Leishmania* in complex with inhibitors present in the PDB show binding to *L*. *infantum* (*Li*) TR of a diarylpirrole-based inhibitor (PDB code: 4APN)[23] and of 6-(sec-butoxy)-2-((3-chlorophenyl)thio)pyrimidin-4-amine (RDS777) (PDB code: 5EBK)[18].

### Activity against *Leishmania infantum* promastigote assays

**Fig 10:**
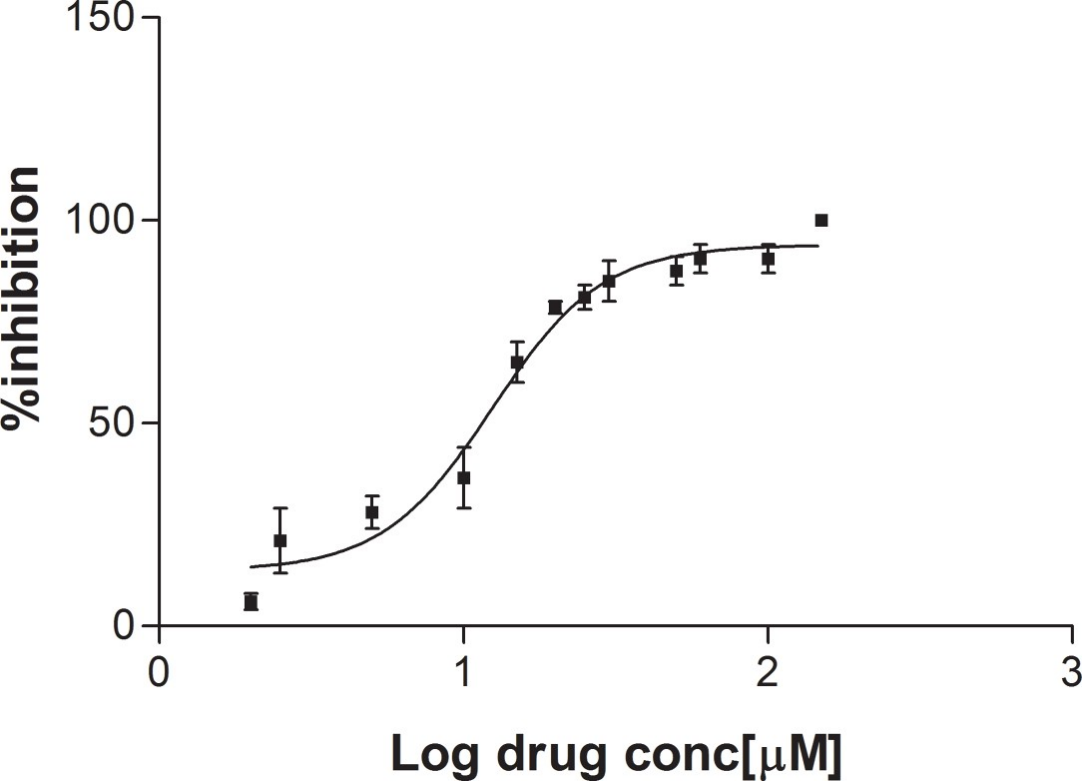
% Inhibition calculated on *L*. *infantum* promastigote stage after treatment with various concentrations of compound 3. The data are expressed as mean ± standard error of two independent experiments.

Compound **3** induced a dose-dependent anti-proliferative effect on *L*. *infantum* promastigotes. Seventy-two hours after treatment, it induced 100% promastigote mortality up to 100 μM, whereas lower drug concentrations induced a dose-dependent anti-proliferative effect on *L*. *infantum* promastigotes. Compound **3** showed IC_50_ of 12.44 ±1.09 μM [95% CI: 10.20–32.64 μM] (**Fig 10**).

## Discussion

The goal of this work was the identification of *Leishmania* TR inhibitors by HTS of a collection of over 100K small molecules. A new luminescence-based TR activity assay was developed in order to allow for robust and reliable testing. The assay was optimized for TS_2_ and NADPH concentrations before it was used to screen the entire collection of 120,000 compounds. The screening effort led to the identification of several hit molecules from which one series was selected for follow-up due the presence of analogs with activity in the micromolar range together with structures judged suitable for further optimization. The prototype compound **3** was fully characterized with respect to its binding and competition with the TR substrate or cofactor and was found to be a reversible and NADPH competitive inhibitor. The low-resolution X-ray structure of the LiTR-compound **3** complex allowed us to identify the binding site of compound **3** (**Fig 8B**). Although the ligand is not completely visible in the structure, the structural information was sufficient to allow us to identify a new ligand binding site in TR that is not conserved in the human homologs hGR and hTrxR1.

As shown in **Fig 9B**, compound **3** is bound to the NADPH cavity entrance forming several weak electrostatic interactions with the protein residues lining its binding site, namely Arg228, Gly197, Tyr221, Asn254 and Arg222 (Fig 8C), thereby impeding the entrance of the substrate in the binding site. The ligand binding causes a significant displacement of the two arginine residues (Arg 222 and Arg 228) that in the NADPH bound TR are involved in the binding of the adenine ribose phosphate moiety (**Figs 9B and S6**). This result explains the competition between compound **3** and NADPH observed biochemically.

The binding of compound **3** to the protein causes a local conformational change involving Arg228 and Arg222 as shown in Fig 9A. This local conformational change induced by inhibitor binding may account for the presence of a biphasic dissociation phase, as shown by SPR assays.

The compound **3** binding site identified by the X-ray structure is unique, since it is present in TR but not in the human homologs hGR and hTxrR1. In particular, the residues Arg235, Tyr221, Gly223, Asn254 important for the ligand binding and the plasticity of the site are substituted by Ser231, Ile217, His219, Gln250 in hGR and Ala233, Val220, Ser222, Val252 in TrxTR1, respectively (**Figs 9C and S7**).

While testing of this compound class on other human NADPH dependent enzymes will be necessary, the initial observation that compound **3** is selective for TR with respect to hGR, together with the unique nature of the its TR binding site, allows us to speculate that NADPH competition for human NADPH binding proteins ought not be an issue for this compound class. Finally, compound **3** showed a dose-dependent anti-proliferative effect on *L*. *infantum* promastigotes at micromolar concentrations suggesting that the compound is indeed able to reach its target at the parasite levels.

Although we expect that at these mid to high concentrations there may be more than one phenomenon contributing to the anti-proliferative effect of compound **3**, based on the presented data, TR inhibition is likely to be one major contributor.

## Supporting information

S1 Table Biological data for (active) follow-up compounds from class i) that are structurally related to compound 3. (DOCX)

S1 Fig NADPH-Glo time course for signal stability determination. The stability of the NADPH-Glo signal was verified in the absence or presence of TR (100 pM) for 60 min. The TS_2_ concentration was 15 μM while the NADPH concentration was 12.5 μM. The results are reported as fold increase with respect to time 5 minutes (i.e. the shortest time possible at which the detection was made). The luminescence signal reaches its steady state after 30 minutes incubation at RT in agreement with the NADPH-Glo manual.
(DOCX)

S2 Fig Reducing substances interference with the NADPH-Glo signal. The effect of reducing agents (DTT and GSH) on the luminescence output of the NADPH-Glo reaction was determined in presence of NADPH concentrations from 3.12 to 50 μM. The signal was detected after 30 minutes at RT. Results are reported as fold variation with respect to the buffer (no reducing agents) control.
(DOCX)

S3 Fig Auranofin interference with DTNB assay. A serial dilution of AF was incubated with TR before (solid circle) and after (open circle) the TR reaction took place. The signal was then developed by the addition of DTNB and normalized between the no-TR (100%) and DMSO (0%) signals. The addition of AF, after the TR reaction was stopped (open circles and fitting), resulted in a dose-dependent decrease of the signal, suggesting that AF at least partially captures the produced T(SH)_2_, thus interfering with the DTNB detection.
(DOCX)

S4 Fig ^1^H NMR spectrum for compound 3. (DOCX)

S5 Fig Compound 3 inhibition curve as determined by DTNB assay and luminescence-based assay. (DOCX)

S6 Fig Superimposition between compound 3-TR complex (in magenta), TR in oxidized state (PDB code: 2JK6) (in blue) and TR in reduced state complexed with NADPH (PDB code: 2W0H). The two Arginine residues (Arg222 and Arg228) involved in the ligand binding are indicated and depicted as sticks. The picture was obtained using PyMOL (The PyMOL Molecular Graphics System, Version 2.0 Schrödinger, LLC.).
(DOCX)

S7 Fig Superimposition between compound 3-TR complex (in magenta) and human Thioredoxin reductase 1 (hTrxR1) (in green) (PDB code: 2CFY). The residues involved in the ligand binding are indicated (the residues of the compound **3**-TR complex on the left side and the residues of human TrxR1 on the right side) and depicted as sticks. The picture was obtained using PyMOL (The PyMOL Molecular Graphics System, Version 2.0 Schrödinger, LLC.).
(DOCX)
