# Supplementary material for: Identification and binding mode of a novel Leishmania Trypanothione reductase inhibitor from high throughput screening
Source: PLoS Negl Trop Dis. 2018 Nov 26;12(11):e0006969. doi: 10.1371/journal.pntd.0006969 (PMC6283646; doi:10.1371/journal.pntd.0006969)
Supplement: S1 Table — (DOCX) [file pntd.0006969.s001.docx]

**S4 Table**. Biological data for (active) follow-up compounds from class i) that are structurally related to compound **3.**

| **ID** | **Structure** | **TR assay IC_50_** (µM) n=3 | **hGR assay IC_50_** (µM) n=3 | **MS [M+H]^+^ Found** | **^a^Purity (%)** |
| --- | --- | --- | --- | --- | --- |
| 2 |  | 13.28 ± 2.69 | 13.91 ± 3.50 | 336 | 97 |
| 3 |  | 7.52 ± 2.53 | >85 | 414 | 96 |
| 4 |  | 23.12 ± 2.66 | >85 | 254 |  |
| 5 |  | 31.34 ± 1.33 | >85 | 271 |  |
| 6 |  | 9.46 ± 1.56 | 31 ± 2.33 | 228 | 96 |
| 7 |  | 28.93 ± 10.30 | >85 | 310 | 99 |
| 8 |  | 28.87 ± 5.45 | >85 | 382 | 99 |
| 9 |  | 4.33 ± 1.96 | >85 | 206 | 99 |

^a^Purity was assessed by UPLC/MS using UV detection (diode array) as described in the experimental section of the main manuscript.
