# Supplementary material for: Identification and binding mode of a novel Leishmania Trypanothione reductase inhibitor from high throughput screening
Source: PLoS Negl Trop Dis. 2018 Nov 26;12(11):e0006969. doi: 10.1371/journal.pntd.0006969 (PMC6283646; doi:10.1371/journal.pntd.0006969)
Supplement: S1 Fig — The stability of the NADPH-Glo signal was verified in the absence or presence of TR (100 pM) for 60 min. The TS2 concentration was 15 μM while the NADPH concentration was 12.5 μM. The results are reported as fold increase with respect to time 5 minutes (i.e. the shortest time possible at which the detection was made). The luminescence signal reaches its steady state after 30 minutes incubation at RT in agreement with the NADPH-Glo manual. (DOCX) [file pntd.0006969.s002.docx]

**S1 Figure:** NADPH glo time course for signal stability determination

The stability of the NADPH-Glo signal was verified in the absence or presence of TR (100 pM) for 60 min. The TS2 concentration was 15 µM while the NADPH concentration was 12.5 µM. The results are reported as fold increase with respect to time 5 minutes (i.e. the shortest time possible at which the detection was made). The luminescence signal reaches it steady state after 30 minutes incubation at RT in agreement with the NADPH-Glo manual.
