# Supplementary material for: Identification and binding mode of a novel Leishmania Trypanothione reductase inhibitor from high throughput screening
Source: PLoS Negl Trop Dis. 2018 Nov 26;12(11):e0006969. doi: 10.1371/journal.pntd.0006969 (PMC6283646; doi:10.1371/journal.pntd.0006969)
Supplement: S2 Fig — The effect of reducing agents (DTT and GSH) on the luminescence output of the NADPH-Glo reaction was determined in presence of NADPH concentrations from 3.12 to 50 μM. The signal was detected after 30 minutes at RT. Results are reported as fold variation with respect to the buffer (no reducing agents) control. (DOCX) [file pntd.0006969.s003.docx]

**S2 Figure:** reducing substances interference with the NADPH Glo signal

The effect of reducing agents (DTT and GSH) on the luminescence output of the NADPH-Glo reaction was determined in presence of NADPH concentrations from 3.12 to 50 µM. the signal was detected after 30 minutes at RT. Results are reported as fold variation with respect to the buffer (no reducing agents) control.
