# Supplementary material for: Identification and binding mode of a novel Leishmania Trypanothione reductase inhibitor from high throughput screening
Source: PLoS Negl Trop Dis. 2018 Nov 26;12(11):e0006969. doi: 10.1371/journal.pntd.0006969 (PMC6283646; doi:10.1371/journal.pntd.0006969)
Supplement: S3 Fig — A serial dilution of AF was incubated with TR before (solid circle) and after (open circle) the TR reaction took place. The signal was then developed by the addition of DTNB and normalized between the no-TR (100%) and DMSO (0%) signals. The addition of AF, after the TR reaction was stopped (open circles and fitting), resulted in a dose-dependent decrease of the signal, suggesting that AF at least partially captures the produced T(SH)2, thus interfering with the DTNB detection. (DOCX) [file pntd.0006969.s004.docx]

**S3 Figure**. Auranofin interference with DTNB assay

A serial dilution of AF was incubated with TR before (solid circle) and after (open circle) the TR reaction took place. The signal was then developed by the addition of DTNB and normalized between the no-TR (100%) and DMSO (0%) signals. The addition of AF, after the TR reaction was stopped (open circles and fitting), resulted in a dose-dependent decrease of the signal, suggesting that AF at least partially captures the produced T(SH)_2_, thus interfering with the DTNB detection.
