# Supplementary figures and images for: Identification and binding mode of a novel Leishmania Trypanothione reductase inhibitor from high throughput screening
Source: PLoS Negl Trop Dis. 2018 Nov 26;12(11):e0006969. doi: 10.1371/journal.pntd.0006969 (PMC6283646; doi:10.1371/journal.pntd.0006969)

**S5 Figure.** 1H NMR spectrum for compound **3**


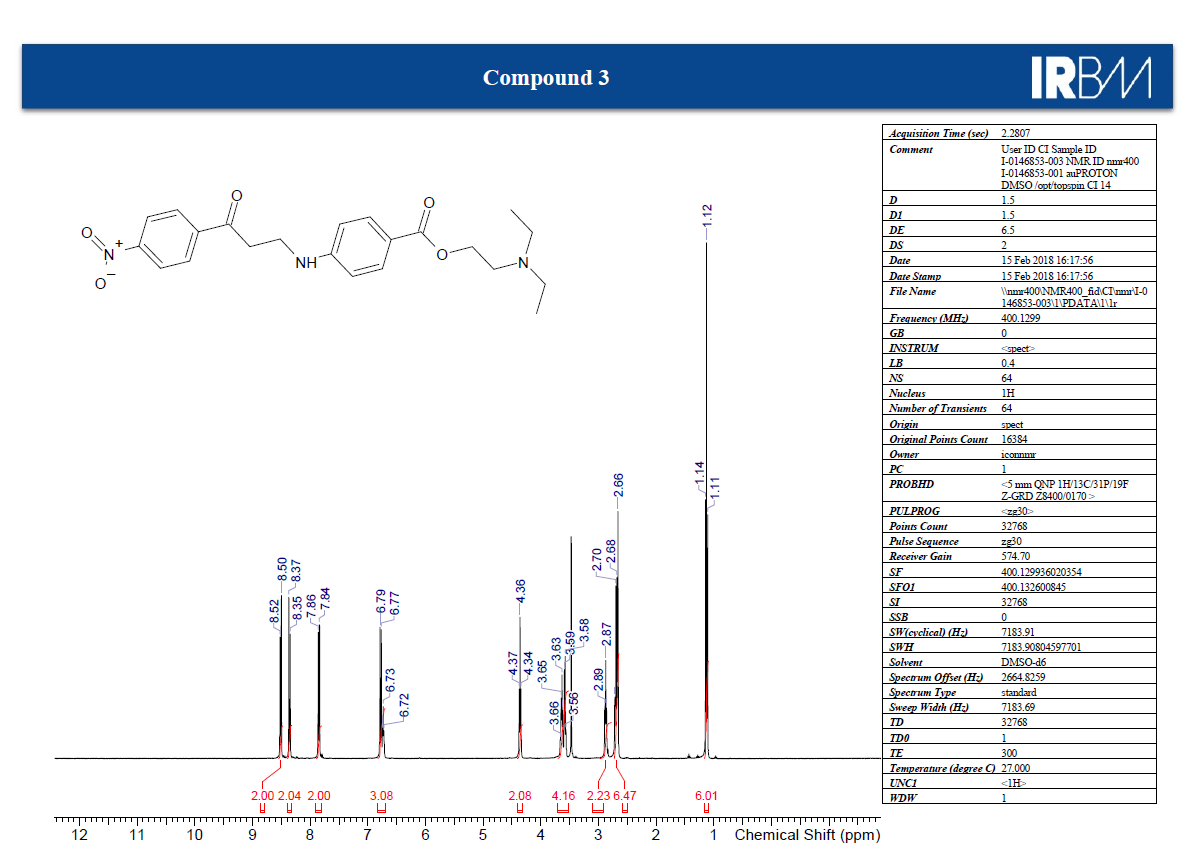

Supplement: S4 Fig — (DOCX) [file pntd.0006969.s005.docx]
