# Supplementary material for: Identification and binding mode of a novel Leishmania Trypanothione reductase inhibitor from high throughput screening
Source: PLoS Negl Trop Dis. 2018 Nov 26;12(11):e0006969. doi: 10.1371/journal.pntd.0006969 (PMC6283646; doi:10.1371/journal.pntd.0006969)
Supplement: S5 Fig — (DOCX) [file pntd.0006969.s006.docx]

**S6 Figure**. Compound **3** inhibition curve as determined by DTNB and luminescence based assay

**DTNB** IC_50_ : 4.0 ± 0.9 μM

**Luminescence assay** IC_50_ : 7.5 ± 2.5 μM.
