# Supplementary material for: Identification and binding mode of a novel Leishmania Trypanothione reductase inhibitor from high throughput screening
Source: PLoS Negl Trop Dis. 2018 Nov 26;12(11):e0006969. doi: 10.1371/journal.pntd.0006969 (PMC6283646; doi:10.1371/journal.pntd.0006969)
Supplement: S6 Fig — The two Arginine residues (Arg222 and Arg228) involved in the ligand binding are indicated and depicted as sticks. The picture was obtained using PyMOL (The PyMOL Molecular Graphics System, Version 2.0 Schrödinger, LLC.). (DOCX) [file pntd.0006969.s007.docx]

**S7 Figure:** Superimposition between compound **3**-TR complex (in magenta), TR in oxidized state (PDB code: 2JK6) (in blue) and TR in reduced state complexed with NADPH (PDB code: 2W0H). The two Arginine residues (Arg222 and Arg228) involved in the ligand binding are indicated and depicted as sticks. The picture was obtained using PyMOL (The PyMOL Molecular Graphics System, Version 2.0 Schrödinger, LLC.)

**
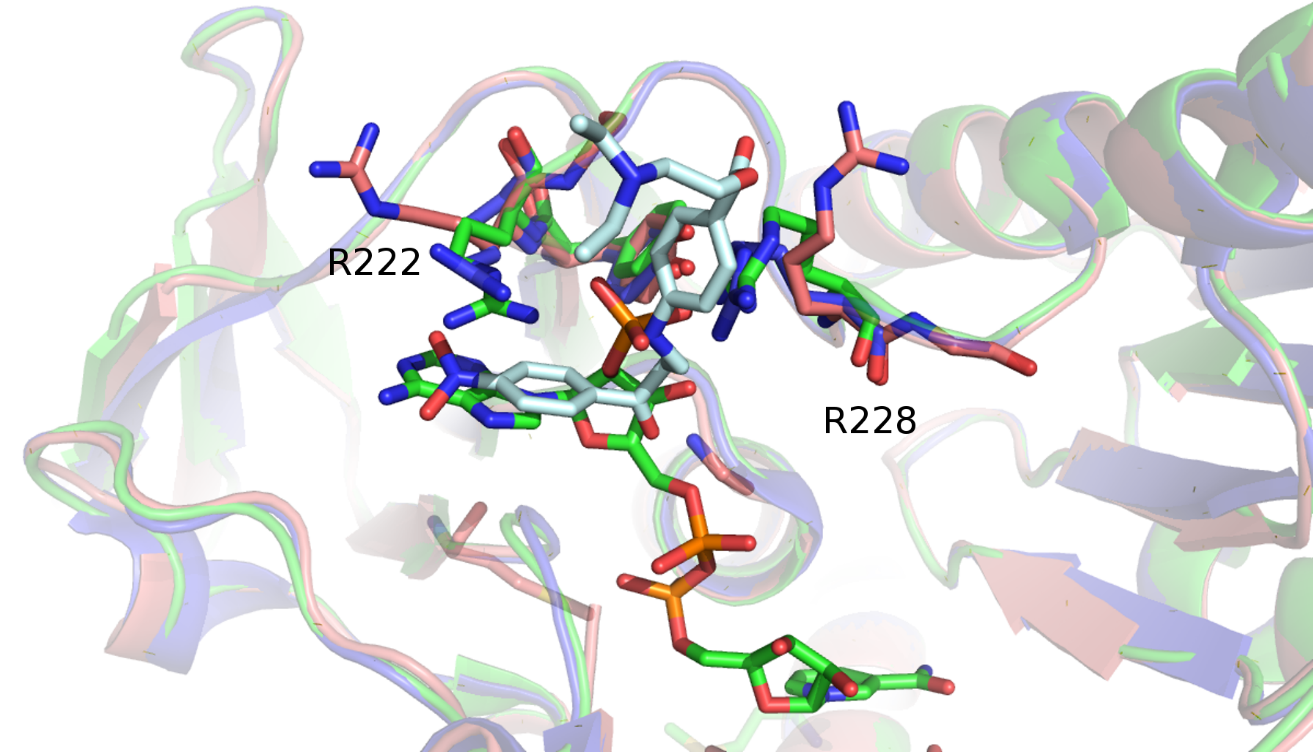
**
