# Supplementary material for: Identification and binding mode of a novel Leishmania Trypanothione reductase inhibitor from high throughput screening
Source: PLoS Negl Trop Dis. 2018 Nov 26;12(11):e0006969. doi: 10.1371/journal.pntd.0006969 (PMC6283646; doi:10.1371/journal.pntd.0006969)
Supplement: S7 Fig — The residues involved in the ligand binding are indicated (the residues of the compound 3-TR complex on the left side and the residues of human TrxR1 on the right side) and depicted as sticks. The picture was obtained using PyMOL (The PyMOL Molecular Graphics System, Version 2.0 Schrödinger, LLC.). (DOCX) [file pntd.0006969.s008.docx]

**S8 Figure:** Superimposition between compound **3**-TR complex (in magenta) and human Thioredoxin reductase 1 (hTrxR1) (in green) (PDB code: 2CFY). The residues involved in the ligand binding are indicated (the residues of the compound **3**-TR complex on the left side and the residues of human TrxR1 on the right side) and depicted as sticks. The picture was obtained using PyMOL (The PyMOL Molecular Graphics System, Version 2.0 Schrödinger, LLC.)

**
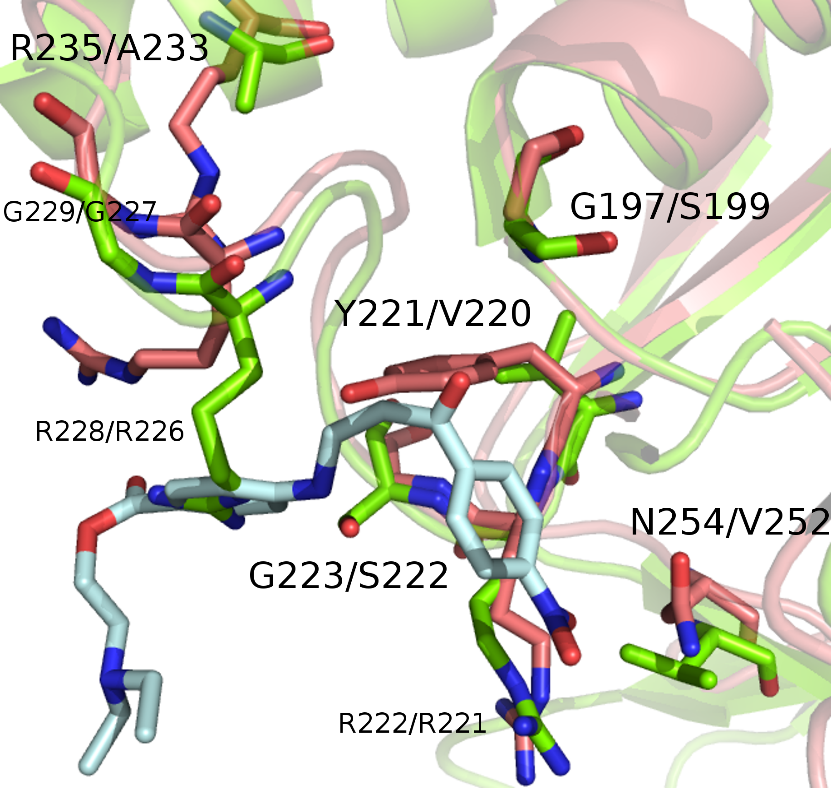
**
